# Supplementary material for: Comparative physiological study of sea cucumbers from eastern waters of United States
Source: PLoS One. 2023 Oct 30;18(10):e0293481. doi: 10.1371/journal.pone.0293481 (PMC10615258; doi:10.1371/journal.pone.0293481)

PLOS ONE

Comparative Physiological Study of Sea Cucumbers from Eastern Waters of United States

--Manuscript Draft--

**Manuscript Number:**	PONE-D-23-17088
**Article Type:**	Research Article
**Full Title:**	Comparative Physiological Study of Sea Cucumbers from Eastern Waters of United States
**Short Title:**	Physiological Study of Sea Cucumber
**Corresponding Author:**	Ahmed Mustafa, Ph.D.<br>Purdue University Fort Wayne<br>Fort Wayne, Indiana UNITED STATES
**Keywords:**	Sea cucumbers, Cucumaria frondosa, Isostychopus badionotus, and Pentacta pygmaea, Physiological and Immunological properties
**Abstract:**	Sea cucumbers, belonging to the phylum Echinodermata, are known to possess valuable<br>bioactive compounds that have medicinal properties. In several countries, such as Korea, China, and Japan, they are cultured in the aquaculture industries for food and medicinal purposes. To find the species with better physiological and immunological properties, we compared the physiological and immunological parameters of three species of sea cucumbers from the waters of the eastern United States namely, Cucumaria frondosa (C. frondosa), Isostychopus badionotus (I. badionotus), and Pentacta pygmaea (P. Pygmaea). When four different cells: phagocytic, red spherule, white spherule, and vibratile cells were counted, C. frondosa illustrated the highest concentrations of phagocytic cells, white spherule cells, and vibratile cells, compared to the two other species ($P<0.05$). Due to its high phagocytic cell concentration, the highest phagocytic capacity was seen in C. frondosa although it was not statistically significant ($P>0.05$). Apart from this, we also observed that C. frondosa had the highest total cell count ($P<0.05$). We also measured coelomic protein concentration and C. frondosa had the highest concentration of coelomic protein ($P<0.05$) among the three species. Lastly, when lysozyme activity was measured, the highest activity was also observed in C. frondosa ($P<0.05$). Looking at the results, we concluded that C. frondosa is the better species with immunological and physiological properties with the potential to be reared in the aquaculture system for use in the food and biomedicine industries.
**Order of Authors:**	Eaint Honey Aung Win
	Ahmed Mustafa, Ph.D.
	Sinthia Mumu
	Nahian Fahim
	kusum Parajuli
	Rebecca Palu
	Elliott Blumenthal
**Additional Information:**	
**Question**	**Response**
**Financial Disclosure**<br><br>Enter a financial disclosure statement that describes the sources of funding for the work included in this submission. Review the submission guidelines for detailed	The author(s) received no specific funding for this work.

Powered by Editorial Manager® and ProduXion Manager® from Aries Systems Corporation

requirements. View published research articles from *PLOS ONE* for specific examples.<br><br>This statement is required for submission and **will appear in the published article** if the submission is accepted. Please make sure it is accurate.<br><br>**Unfunded studies**<br>Enter: *The author(s) received no specific funding for this work.*<br><br>**Funded studies**<br>Enter a statement with the following details:<br>• Initials of the authors who received each award<br>• Grant numbers awarded to each author<br>• The full name of each funder<br>• URL of each funder website<br>• Did the sponsors or funders play any role in the study design, data collection and analysis, decision to publish, or preparation of the manuscript?<br>• **NO** - Include this sentence at the end of your statement: *The funders had no role in study design, data collection and analysis, decision to publish, or preparation of the manuscript.*<br>• **YES** - Specify the role(s) played.<br><br>* typeset	
**Competing Interests**<br><br>Use the instructions below to enter a competing interest statement for this submission. On behalf of all authors, disclose any competing interests that could be perceived to bias this work—acknowledging all financial support and any other relevant financial or non-financial competing interests.<br><br>This statement is required for submission and **will appear in the published article** if the submission is accepted. Please make sure it is accurate and that any funding sources listed in your Funding Information later in the submission form are also declared in your Financial Disclosure	The authors have declared that no competing interests exist.

statement.<br><br>View published research articles from *PLOS ONE* for specific examples.<br><br>**NO authors have competing interests**<br><br>Enter: *The authors have declared that no competing interests exist.*<br><br>**Authors with competing interests**<br><br>Enter competing interest details beginning with this statement:<br><br>*I have read the journal's policy and the authors of this manuscript have the following competing interests: [insert competing interests here]*<br><br>* typeset	
**Ethics Statement**<br><br>Enter an ethics statement for this submission. This statement is required if the study involved:<br><br>• Human participants<br>• Human specimens or tissue<br>• Vertebrate animals or cephalopods<br>• Vertebrate embryos or tissues<br>• Field research<br><br>Write "N/A" if the submission does not require an ethics statement.<br><br>General guidance is provided below. Consult the submission guidelines for detailed instructions. **Make sure that all information entered here is included in the Methods section of the manuscript.**	All animals were taken care of ethically. No formal ‘IACUC- Animal Care Protocol Approval’ was needed as these animals were invertebrates.

Powered by Editorial Manager® and ProduXion Manager® from Aries Systems Corporation

**Format for specific study types**<br><br>**Human Subject Research (involving human participants and/or tissue)**<br>• Give the name of the institutional review board or ethics committee that approved the study<br>• Include the approval number and/or a statement indicating approval of this research<br>• Indicate the form of consent obtained (written/oral) or the reason that consent was not obtained (e.g. the data were analyzed anonymously)<br><br>**Animal Research (involving vertebrate animals, embryos or tissues)**<br>• Provide the name of the Institutional Animal Care and Use Committee (IACUC) or other relevant ethics board that reviewed the study protocol, and indicate whether they approved this research or granted a formal waiver of ethical approval<br>• Include an approval number if one was obtained<br>• If the study involved *non-human primates*, add *additional details* about animal welfare and steps taken to ameliorate suffering<br>• If anesthesia, euthanasia, or any kind of animal sacrifice is part of the study, include briefly which substances and/or methods were applied<br><br>**Field Research**<br><br>Include the following details if this study involves the collection of plant, animal, or other materials from a natural setting:<br>• Field permit number<br>• Name of the institution or relevant body that granted permission	
**Data Availability**<br><br>Authors are required to make all data underlying the findings described fully available, without restriction, and from the time of publication. PLOS allows rare exceptions to address legal and ethical concerns. See the PLOS Data Policy and FAQ for detailed information.	Yes - all data are fully available without restriction

A Data Availability Statement describing where the data can be found is required at submission. Your answers to this question constitute the Data Availability Statement and **will be published in the article**, if accepted.<br><br>**Important:** Stating 'data available on request from the author' is not sufficient. If your data are only available upon request, select 'No' for the first question and explain your exceptional situation in the text box.<br><br>Do the authors confirm that all data underlying the findings described in their manuscript are fully available without restriction?	
**Describe where the data may be found in full sentences. If you are copying our sample text, replace any instances of XXX with the appropriate details.**<br><br>• If the data are **held or will be held in a public repository**, include URLs, accession numbers or DOIs. If this information will only be available after acceptance, indicate this by ticking the box below. For example: *All XXX files are available from the XXX database (accession number(s) XXX, XXX.).*<br>• If the data are all contained **within the manuscript and/or Supporting Information files**, enter the following: *All relevant data are within the manuscript and its Supporting Information files.*<br>• If neither of these applies but you are able to provide **details of access elsewhere**, with or without limitations, please do so. For example:<br><br>*Data cannot be shared publicly because of [XXX]. Data are available from the XXX Institutional Data Access / Ethics Committee (contact via XXX) for researchers who meet the criteria for access to confidential data.*<br><br>*The data underlying the results presented in the study are available from (include the name of the third party*	All relevant data are within the manuscript and its Supporting Information files.

and contact information or URL).<br>• This text is appropriate if the data are owned by a third party and authors do not have permission to share the data.<br><br>* typeset	
Additional data availability information:	

Manuscript Click here to access/download;Manuscript;Win_manuscript.docx

Comparative Physiological Study of Sea Cucumbers from Eastern Waters of United States

Eaint Honey Aung Win, Sinthia Mumu, Nahian Fahim, Kusum Parajuli, Elliott Blumenthal, Rebecca Palu and Ahmed Mustafa*

Department of Biological Sciences, Purdue University Fort Wayne
2101 East Coliseum Boulevard, Fort Wayne, IN 46805, USA

***Correspondence:**
Dr. Ahmed Mustafa
Department of Biological Sciences, Purdue University Fort Wayne,
2101 E. Coliseum Blvd., Fort Wayne, IN 46805, USA
Email: mustafaa@pfw.edu

Abstract:

Sea cucumbers, belonging to the phylum Echinodermata, are known to possess valuable bioactive compounds that have medicinal properties. In several countries, such as Korea, China, and Japan, they are cultured in the aquaculture industries for food and medicinal purposes. To find the species with better physiological and immunological properties, we compared the physiological and immunological parameters of three species of sea cucumbers from the waters of the eastern United States namely, *Cucumaria frondosa* (*C. frondosa*), *Isostychopus badionotus* (*I. badionotus*), and *Pentacta pygmaea* (*P. Pygmaea*). When four different cells: phagocytic, red spherule, white spherule, and vibratile cells were counted, *C. frondosa* illustrated the highest concentrations of phagocytic cells, white spherule cells, and vibratile cells, compared to the two other species ($P<0.05$). Due to its high phagocytic cell concentration, the highest phagocytic capacity was seen in *C. frondosa* although it was not statistically significant ($P>0.05$). Apart from this, we also observed that *C. frondosa* had the highest total cell count ($P<0.05$). We also measured coelomic protein concentration and *C. frondosa* had the highest concentration of coelomic protein ($P<0.05$) among the three species. Lastly, when lysozyme activity was measured, the highest activity was also observed in *C. frondosa* ($P<0.05$). Looking at the results, we concluded that *C. frondosa* is the better species with immunological and physiological properties with the potential to be reared in the aquaculture system for use in the food and biomedicine industries.

Keywords: Sea cucumbers, *Cucumaria frondosa*, *Isostychopus badionotus*, and *Pentacta pygmaea*, Physiological and Immunological properties

Introduction

Sea cucumbers, also known as Holothurians, are invertebrates that belong to the phylum Echinodermata [1]. These animals, unlike others, have a unique elongated body structure that is supported by a hydrostatic skeleton, where the body muscle is shaped by a fluid-filled cavity, the coelom [2]. In Asian countries, species such as *Stichopus hermanni*, *Thelenota ananas*, *Thelenota anax*, *Holothuria fuccogilva*, and *Actinopyga mauritiana* are used as a functional food and traditional medication [3]. In recent studies, it was discovered that sea cucumbers possess bioactive compounds that have 16 valuable medicinal properties [4]. According to Xu et al. (2018), polysaccharides, triterpene glycosides, phenols, and lipids can be isolated from the invertebrate [5]. Several studies have shown that these compounds have anti-cancer, anti-inflammatory, anti-microbial, anti-angiogenic, anti-hypertension, anti-hyperglycemic, anti-oxidant, and immunomodulatory activities [6]. Due to this, sea cucumbers are considered to have the potential to be reared in the aquaculture system for use in the food and biomedicine industries.

Although some research has been conducted on several species of sea cucumbers around the world, not many studies have focused on the species of sea cucumbers in the waters of the United States. One of the species, the Orange-footed Sea cucumber, also known as *C. frondosa* can be found in the cold waters of the Atlantic Ocean in the United States. This species can be found in the benthic areas of the water, and they have an elongated leathery body [6,7]. According to a previous study, *C. frondosa* has the ability to grow up to 40-50cm in length and 10-15cm in width [6]. Due to its medicinal and nutritional benefits, *C. frondosa* is seen as a species to further culture in the aquaculture industry [8]. *I. badionotus* is a type of sea cucumber that is also called the chocolate chip sea cucumber and it can be found in the Gulf of Mexico [1,9]. According to Acosta et al. (2021), this species can be found primarily on rocky bottoms

between the cracks that are in 2.5m depth of water and they tend to have an average size and weight of 324mm and 628g respectively [10]. In recent studies, medicinal values such as anti-coagulant and anti-inflammatory properties have been found in this species, and in several countries, the animal is considered edible for nutritional purposes [11,12]. The last species in this study is *P. pygmaea* and it resides in the water of the Gulf of Mexico [13]. This species is brown in color and is considered to be small in size (4-6cm) with ossicles in its body wall that makes it stiffer than other sea cucumbers [14]. Although this species is not known to be edible, it has been found to have bioactive compounds with the potential to treat SARS-CoV-2 [15].

In many animals, it has been found that stress-induced changes can suppress the immune response in an animal [16]. For animals in an aquaculture system, one of the stressors that the animals often encounter is handling stress and this can reduce the quality of the species [17,18]. As a result, before culturing a species in the aquaculture system, it is important to discern the ability of the sea cucumber to mitigate stress without affecting the overall health and quality of the animal, or its nutritional and medicinal benefits. The immune responses of sea cucumbers depend on the coelomocytes that are in the coelomic fluid [19]. During stressful conditions and pathogenic exposures, these cells participate in maintaining homeostasis and eliminating pathogens [19].

As the sea cucumbers encounter stress or pathogens, the invertebrate undergoes cellular responses to maintain homeostasis and overcome the disease state [20]. In the coelomic fluid of sea cucumbers, there are four major types of coelomocytes, and they are, red spherule cells, white spherule cells, phagocytic cells, and vibratile cells [19]. They all have functions relating to antibacterial activity, inflammation, wound healing, encapsulation, graft rejection, and cytotoxic activity in the body of sea cucumbers [21]. The red and white spherule cells were found to secret

lipase, peroxidase, and serine proteinase resulting in the breakdown of materials after the phagocytosis [22,23]. Phagocytes contain lysosomal enzymes and have the ability to ingest and destroy unwanted organisms or particles that they encounter [24]. Vibratile cells are highly motile and known to assist in the circulation of the coelomic fluid [25,26]. The vibratile cells also can degranulate during the clotting process in the animal [25].

Although studies have been conducted on the medicinal and nutritional benefits of sea cucumbers, they have only been focused on species that reside in the East Asian and Middle Eastern countries [2,3,27-29]. Due to this, it is important to explore the medicinal properties of sea cucumber species in other parts of the world. One of the areas that were not studied extensively for sea cucumber species and their medicinal benefit is the United States. In this experiment, we study the immunological and physiological properties of coelomic fluid from three sea cucumber species collected from the waters of the Eastern United States: *Cucumaria frondosa* (*C. frondosa*), *Isostychopus badionotus* (*I. badionotus*), and *Pentacta pygmaea* (*P. Pygmaea*). The physiological parameters measured in this study will tell us about the overall health of the sea cucumbers.

Material and methods

Collection of Coelomic Fluid and Tissue

Three species of sea cucumbers, *Cucumaria frondosa*, *Isostychopus badionotus*, and *Pentacta pygmaea* were purchased from Gulf of Maine Inc (Pembroke, Maine) and Gulf Specimen Marine Lab (Panacea, Florida) respectively and reared in the aquaculture lab of Purdue University Fort Wayne. Coelomic fluid was collected through midsagittal dissection with a syringe after the animals were euthanized (N=6 for each species). The coelomic fluid was immediately kept on ice for analysis of coelomocytes, coelomic protein level, lysozyme activity, and phagocytic

capacity. The tissues (Body Wall, Viscera, and Tentacle) obtained from these invertebrates were kept at -80◦C after rinsing for future experiments.

Total and Differential Cell Count

After obtaining the coelomic fluid samples, 50µl of coelomic fluid was mixed with 50µl of the anticoagulant, Dipotassium Ethylenediamine Tetraacetate (Sequester-Sol, USA). Then, 25µl of the mixture was loaded into a hemocytometer. Four different types of cells, phagocytic cells - red spherule cells, white spherule cells, and vibratile - were counted to find the differential coelomocyte count (DCC) (Figure 2.1). Then the differential cell counts were summed up to find the total coelomocytes count (TCC). Top left and right 16 squares of the hemocytometer were counted and averaged. To obtain cells per milliliter, the following equation described by Yeh et al., (2004) was used [30]:

$$\frac{Cells}{mL} = \frac{cell\ count}{number\ of\ counted\ corners} \times dilution\ factor\ (2) \times 10^4$$

Total Coelomic Protein

Total coelomic fluid protein was measured using a Protein Refractometer (VEEGEE Scientific Inc. Kirkland, WA). Two to three drops of coelomic fluid without anticoagulant were added to the surface of the prism of the calibrated refractometer. Afterward, coelomic protein (g/100ml) was read holding the refractometer under the light.

Lysozyme Activity Assay

Before initiating the lysozyme assay, a suspension of *Micrococcus lysodeikticus* was made at a concentration of 0.2mg/ml using 0.05M (pH =6.2) sodium phosphate buffer. 25µl of collected coelomic fluid without anticoagulant was added into a cuvette with 1ml of the bacterial suspension. Then, the absorbance was measured at 1 minute and 5 minutes using a spectrophotometer at 540 nm. Lysozyme activity assay (LAA) was calculated according to the formula described by Mumu & Mustafa (2022)[31]:

$$LAA = \frac{(final\ absorbance -\ initial\ absorbance)}{total\ elapsed\ time\ (minute)}$$

Phagocytic Capacity

50µl of coelomic fluid was mixed with 50µl of the anticoagulant. Then, to a double cytoslide microscope slide, 50µl was pipetted into each circle [32]. The slide was incubated for 90-120 minutes at room temperature. After the first incubation, 50µl of formalin-killed bacteria (*Bacillus megaterium*) was added to each circle of the glass slide and was incubated again for 60 minutes at room temperature. When incubation was done, the slide was washed with phosphate buffer saline (PBS) for 1 minute. Then, it was fixed with methanol for 1 minute, stained with Wright-Giensa stain for 20 seconds, and rinsed with PBS. The slide was air-dried and counted under the microscope. Positive (≥5 engulfed bacteria) and negative phagocytic cells at a location were recorded to find the percent of phagocytic capacity [25].

Result

From the coelomic fluid obtained from each species, four different cell types were counted: phagocytic, red spherule, white spherule, and vibratile (Figure 2.2). We found that there was a

total of 127.83±21.11 x 10^4 cells/mL, 27.50±9.41 x 10^4 cells/mL, and 56.00±0 x 10^4 cells/mL of phagocytic cells for *C. frondosa*, *I. badionotus*, and *P. pygmaea* respectively. *C. frondosa,* when compared statistically to *I. badionotus* for phagocytic cells concentration, significant differences were seen between the species ($P<0.05$). On the other hand, when the phagocytic cell concentration of *C. frondosa* was compared to *P. pygmaea,* no statistical significance was found ($P>0.05$). For red spherule cells, there were 14.83±10.52 x 10^4 cells/mL for *C. frondosa*, 58.50±8.96 x 10^4 cells/mL for *I. badionotus*, and 13.00±0 x 10^4 cells/mL for *P. pygmaea*. No significant differences were found among the three species ($P>0.05$). The species, *C. frondosa*, *I. badionotus*, and *P. pygmaea* had 30.83±3.29 x 10^4 cells/mL, 25.50±8.96 x 10^4 cells/mL, and 15.00±0 x 10^4 cells/mL of white spherule respectively. No significant differences in white spherule cell concentrations were found among the groups ($P>0.05$). Lastly, there were 141.67±23.85 x 10^4 cells/mL, 27.50±11.08 x 10^4 cells/mL, and 63.00±0 x 10^4 cells/mL of vibratile cells for *C. frondosa*, *I. badionotus*, and *P. pygmaea* respectively. The concentration of vibratile cells between *C. frondosa* and *I. badionotus* were significantly different ($P<0.05$). When *C. frondosa* was compared to *P. pygmaea*, no statistical significance was found ($P>0.05$).

The total cell count was obtained for each type of species (Figure 2.3). The total cell count concentrations were 315.17±71.65 x 10^4 cells/mL for *C. frondosa*, 139.00±45.09 x 10^4 cells/mL for *I. badionotus,* and 147.00±0 x 10^4 cells/mL for *P. pygmaea*. When the statistical analysis was conducted, the total cell count concentration for *C. frondosa* was significantly different when compared to *I. badionotus* and *P. pygmaea* ($P<0.05$).

Coelomic fluid protein concentration was 1.85±0.09 g/100mL for *C. frondosa*, 2.80±0.07 g/100mL for *I. badionotus*, and 3.58±0.13 g/100mL for *P. pygmaea* (Figure 2.4). Coelomic protein concentrations were all significantly different from one species to another and *C.*

frondosa produced the lowest concentration of coelomic protein compared to *I. badionotus*, and *P. pygmaea* ($P<0.05$).

We also examined the lysozyme activity of the three species to measure the ability of lysozyme present in the coelomic fluid to break down bacterial cell walls over a period (Figure 2.5). We observed that the activities were $2.60 \times 10^{-3} \pm 5.60 \times 10^{-4}$ absorbance/minute for *C. frondosa*, $6.50 \times 10^{-4} \pm 7.4 \times 10^{-5}$ absorbance/minute for *I. badionotus*, and $1.25 \times 10^{-3} \pm 1.50 \times 10^{-4}$ absorbance/minute for *P. pygmaea*. After statistical analysis, lysozyme activity of *C. frondosa* was significantly different when compared to *I. badionotus* and *P. pygmaea* ($P<0.05$). Lastly, we looked at phagocytic capacity as a measure of immunological status (Figure 2.6). The percentages of phagocytic capacities were $85.86 \pm 4.30\%$, $76.25 \pm 3.33\%$, and $69.12 \pm 4.01\%$ respectively for *C. frondosa*, *I. badionotus*, and *P. pygmaea*. No statistical differences were found between the species ($P>0.05$).

Discussion

Based on the results obtained in this project, we were able to find the best species with physiological and immunological parameters. We can look at this by the differential cell count concentrations. Overall, we observed that *C. frondosa* had higher cell counts for all types that were counted, as well as the total cell count. In a study by Jobson et al. (2021), the authors also showed that higher coelomocyte counts were seen in *C. frondosa* when stress was induced [33]. Others have also shown that enhanced concentrations of phagocytes were seen over time as the sea cucumber was induced with a chemical stress [20]. In our result for differential cell count, we also saw that *C. frondosa* had a significantly high number of phagocytic cells, which could indicate a better immune response. Even though the data analysis showed no significant

differences, some phagocytic capacity was seen in *C. frondosa*. Although not all results for differential and total cell counts were significant, we can imply that *C. frondosa* has the highest number of counted immune cells in its coelomic fluid compared to *I. badionotus* and *P. pygmaea*.

During stressful and pathogenic encounters, proteins help maintain homeostasis and fight against pathogens are secreted into the coelomic fluid [34]. We measured the coelomic protein concentrations of the three species and found that *C. frondosa* had the highest concentration of coelomic protein among the three species ($P<0.05$). In a previous study, it has been shown that sea cucumbers produce an increased amount of proteins such as heat shock proteins and lysozymes to maintain homeostasis during stressful situations [35,36]. In addition to the coelomic protein, we also observed significantly different lysozyme activity when C. *frondosa* was compared to *I. badionotus* and *P. pygmaea* ($P<0.05$). We found that C. *frondosa* has the highest lysozyme activity and lysozyme activity was also seen in the coelomic fluid of *C. frondosa* in a previous study [37].

Conclusion

Based on these results, we can conclude that *C. frondosa* has the best immunological and physiological properties among the three species. It is important to find the best species for the aquaculture industry as species need to be able to handle stress and maintain healthy conditions in order to be cultured for nutritional and medicinal purposes.

Funding Information

The author(s) received no specific funding for this work.

Declaration of interest

There was no conflict of interest.

Data availability statement

The data that support the findings of this study are available on request from the corresponding author.

References

1. Toral-Granda, V., & Mercier, A. Sea cucumbers. A global review on fishery and trade. Fisheries and Aquaculture Technical Paper. 2008. Available from: https://www.researchgate.net/publication/262973901

2. Purcell SW, Samyn Y, Conand C. Commercially important sea cucumbers of the world. Rome: Food and Agriculture Organization of the United Nations; 2012.

3. Pangestuti R, Arifin Z. Medicinal and health benefit effects of functional sea cucumbers. Journal of Traditional and Complementary Medicine. 2018;8: 341–351. doi:10.1016/j.jtcme.2017.06.007

4. Bordbar S, Anwar F, Saari N. High-value components and bioactives from sea cucumbers for functional foods—a review. Marine Drugs. 2011;9: 1761–1805. doi:10.3390/md9101761

5. Xu C, Zhang R, Wen Z. Bioactive compounds and biological functions of sea cucumbers as potential functional foods. Journal of Functional Foods. 2018;49: 73–84. doi:10.1016/j.jff.2018.08.009

6. Hossain A, Dave D, Shahidi F. Northern Sea cucumber (*Cucumaria frondosa):* A potential candidate for functional food, nutraceutical, and pharmaceutical sector. Marine Drugs. 2020;18: 274. doi:10.3390/md18050274

7. Holtz EH, MacDonald BA. Feeding behaviour of the sea cucumber *Cucumaria frondosa* (echinodermata: Holothuroidea) in the laboratory and the field: Relationships between Tentacle insertion rate, flow speed, and ingestion. Marine Biology. 2009;156: 1389–1398. doi:10.1007/s00227-009-1179-0

8. Pietrak M, Kim J, Redmond S, Kim Y-D, Yarish C. Culture of Sea Cucumbers in Korea: A guide to Korean methods and the local sea cucumber in the Northeast US. University of Maine; 2014. Available: https://digitalcommons.library.umaine.edu/cgi/viewcontent.cgi?article=1039&context=seagrant_pub

9. Drake VI, Kim E, Nigro HG, Bogantes VE, Janosik AM. The complete mitochondrial genome of the chocolate chip sea cucumber *Isostichopus badionotus* (echinodermata: Holothuroidea). Mitochondrial DNA Part B. 2021;6: 1947–1948. doi:10.1080/23802359.2021.1937365

10. Acosta EJ, Rodríguez-Forero A, Werding B, Kunzmann A. Ecological and reproductive characteristics of holothuroids *Isostichopus badionotus* and *Isostichopus* sp. in Colombia. PLOS ONE. 2021;16. doi:10.1371/journal.pone.0247158

11. Chen S, Hu Y, Ye X, Li G, Yu G, Xue C, et al. Sequence determination and anticoagulant and antithrombotic activities of a novel sulfated Fucan isolated from the sea cucumber *Isostichopus badionotus*. Biochimica et Biophysica Acta (BBA) - General Subjects. 2012;1820: 989–1000. doi:10.1016/j.bbagen.2012.03.002

12. Olivera-Castillo L, Grant G, Kantún-Moreno N, Acevedo-Fernández JJ, Puc-Sosa M, Montero J, et al. Sea cucumber (*Isostichopus badionotus*) body-wall preparations exert anti-inflammatory activity in vivo. PharmaNutrition. 2018;6: 74–80. doi:10.1016/j.phanu.2018.03.002

13. Delgado-Blas, V. H. Spatial and temporal distribution of benthic Polychaeta from the continental shelf of Tamaulipas, Gulf of Mexico. Revista de Biologia Tropical. 2001;49:1, 141–147. 11795142

14. Red footed sea cucumber. Gulf Specimen Marine Lab. Available: https://491.899.myftpupload.com/red-footed-sea-cucumber/

15. Dwivedi R, Samanta P, Sharma P, Zhang F, Mishra SK, Kucheryavy P, et al. Structural and kinetic analyses of holothurian sulfated glycans suggest potential treatment for SARS-COV-2 infection. Journal of Biological Chemistry. 2021;297: 101207. doi:10.1016/j.jbc.2021.101207

16. Adamo SA. The effects of the stress response on immune function in invertebrates: An evolutionary perspective on an ancient connection. Hormones and Behavior. 2012;62: 324–330. doi:10.1016/j.yhbeh.2012.02.012

17. Barton BA, Iwama GK. Physiological changes in fish from stress in aquaculture with emphasis on the response and effects of corticosteroids. Annual Review of Fish Diseases. 1991;1: 3–26. doi:10.1016/0959-8030(91)90019-g

18. Yang M, Li X, Hu F, Ning Y, Tian R, Ding P, et al. Effects of handling stresses on fitness related behaviors of small sea cucumbers apostichopus japonicus: New insights into seed production. Aquaculture. 2022;546: 737321. doi:10.1016/j.aquaculture.2021.737321

19. Ramírez-Gómez F, Aponte-Rivera F, Méndez-Castaner L, García-Arrarás JE. Changes in Holothurian coelomocyte populations following immune stimulation with different molecular patterns. Fish & Shellfish Immunology. 2010;29: 175–185. doi:10.1016/j.fsi.2010.03.013

20. Hamel J-F, Jobson S, Caulier G, Mercier A. Evidence of anticipatory immune and hormonal responses to predation risk in an echinoderm. Scientific Reports. 2021;11. doi:10.1038/s41598-021-89805-0

21. Gross PS, Al-Sharif WZ, Clow LA, Smith LC. Echinoderm immunity and the evolution of the complement system. Developmental & Comparative Immunology. 1999;23: 429–442. doi:10.1016/s0145-305x(99)00022-1

22. Canicattí C, Tschopp J. Holozyme a: One of the serine proteases of *Holothuria polii* coelomocytes. Comparative Biochemistry and Physiology Part B: Comparative Biochemistry. 1990;96: 739–742. doi:10.1016/0305-0491(90)90223-g

23. Johnson PT. The coelomic elements of sea urchins (*Strongylocentrotus*). Histochemie. 1969;17: 213–231. doi:10.1007/bf00309866

24. Dang H, Zhang T, Yi F, Ye S, Liu J, Li Q, et al. Enhancing the immune response in the sea cucumber *Apostichopus japonicus* by addition of Chinese herbs houttuynia cordata thunb as a food supplement. Aquaculture and Fisheries. 2019;4: 114–121. doi:10.1016/j.aaf.2018.12.004

25. Chia F-S, Xing J. Review Article Echinoderm Coelomocytes. Zoological Studies. 1996;35: 231–254.

26. Smith LC, Ghosh J, Buckley KM, Clow LA, Dheilly NM, Haug T, et al. Echinoderm immunity. Advances in Experimental Medicine and Biology. 2010;708: 260–301. doi:10.1007/978-1-4419-8059-5_14

27. Mohamed IN. Optimum dose of sea cucumber (*Stichopus chloronotus*) extract for wound healing. Medicine and Health. 2017;12: 83–89. doi:10.17576/mh.2017.1201.09

28. Sicuro B, Levine J. Sea cucumber in the Mediterranean: A potential species for aquaculture in the Mediterranean. Reviews in Fisheries Science. 2011;19: 299–304. doi:10.1080/10641262.2011.598249

29. Wijesinghe WAJP, Jeon YJ, Ramasamy P, Wahid ME, Vairappan CS. Anticancer activity and mediation of apoptosis in human HL-60 leukaemia cells by edible sea cucumber (*Holothuria edulis*) extract. Food Chemistry. 2013;139: 326–331. doi:10.1016/j.foodchem.2013.01.058

30. Yeh S-T, Liu C-H, Chen J-C. Effect of copper sulfate on the immune response and susceptibility to vibrio alginolyticus in the white shrimp *Litopenaeus vannamei*. Fish & Shellfish Immunology. 2004;17: 437–446. doi:10.1016/j.fsi.2004.04.016

31. Mumu SK, Mustafa A. Modulation of acute stress and immune response in tilapia, *Oreochromis niloticus*, using longevity spinach, *Gynura procumbens* extract, as nutraceuticals. Journal of Immunoassay and Immunochemistry. 2022;43: 678–694. doi:10.1080/15321819.2022.2080558

32. Mustafa A, MacWilliams C, Fernandez N, Matchett K, Conboy GA, Burka JF. Effects of sea lice (*Lepeophtheirus Salmonis* Kröyer, 1837) infestation on macrophage functions in Atlantic salmon (*Salmo salar L.*). Fish & Shellfish Immunology. 2000;10: 47–59. doi:10.1006/fsim.1999.0229

33. Jobson S, Hamel J-F, Hughes T, Mercier A. Cellular, hormonal, and behavioral responses of the holothuroid *Cucumaria frondosa* to environmental stressors. Frontiers in Marine Science. 2021;8. doi:10.3389/fmars.2021.695753

34. Patruno M, Thorndyke MC, Candia Carnevali MD, Bonasoro F, Beesley PW. Growth factors, heat-shock proteins and regeneration in echinoderms. Journal of Experimental Biology. 2001;204: 843–848. doi:10.1242/jeb.204.5.843

35. Meng X-liang, Dong Y-wei, Dong S-lin, Yu S-shan, Zhou X. Mortality of the sea cucumber, *Apostichopus japonicus selenka*, exposed to acute salinity decrease and related physiological responses: Osmoregulation and heat shock protein expression. Aquaculture. 2011;316: 88–92. doi:10.1016/j.aquaculture.2011.03.003

36. Tian Y, Liang X-wang, Chang Y-qing, Song J. Expression of C-type lysozyme gene in sea cucumber (*Apostichopus japonicus*) is highly regulated and time dependent after Salt Stress. Comparative Biochemistry and Physiology Part B: Biochemistry and Molecular Biology. 2015;180: 68–78. doi:10.1016/j.cbpb.2014.10.004

37. Haug T, Kjuul AK, Styrvold OB, Sandsdalen E, Olsen ØM, Stensvåg K. Antibacterial activity in *Strongylocentrotus droebachiensis* (echinoidea), *Cucumaria frondosa* (holothuroidea), and *Asterias Rubens* (Asteroidea). Journal of Invertebrate Pathology. 2002;81: 94–102. doi:10.1016/s0022-2011(02)00153-2

Figure **Error! No text of specified style in document.**.1 Types of coelomocytes in echinoderms; red arrow= red spherule cells, white arrow= white spherule cells, blue arrow= vibratile cells, black arrow = phagocytic cells [26].

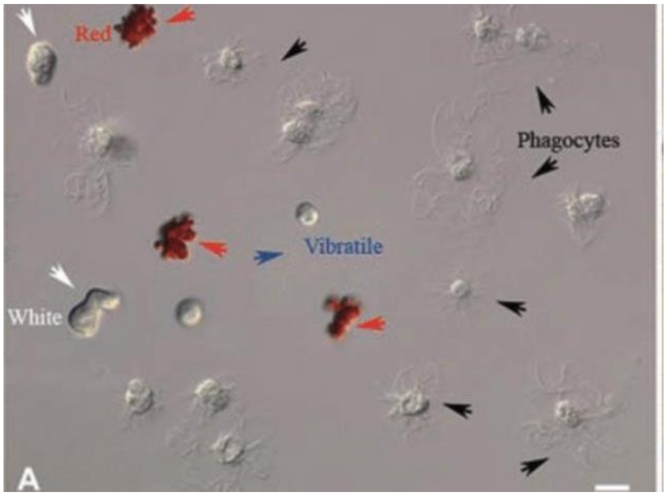

Figure **Error! No text of specified style in document.**.2 Differential cell count (cells/mL) of *Cucumaria frondosa* (*C. frondosa*), *Isostychopus badionotus* (*I. badionotus*), and *Pentacta*

pygmaea (*P. pygmaea*). The cell concentrations illustrated are averaged. Different alphabets represent significantly different concentrations among the species for each cell type (P<0.05).

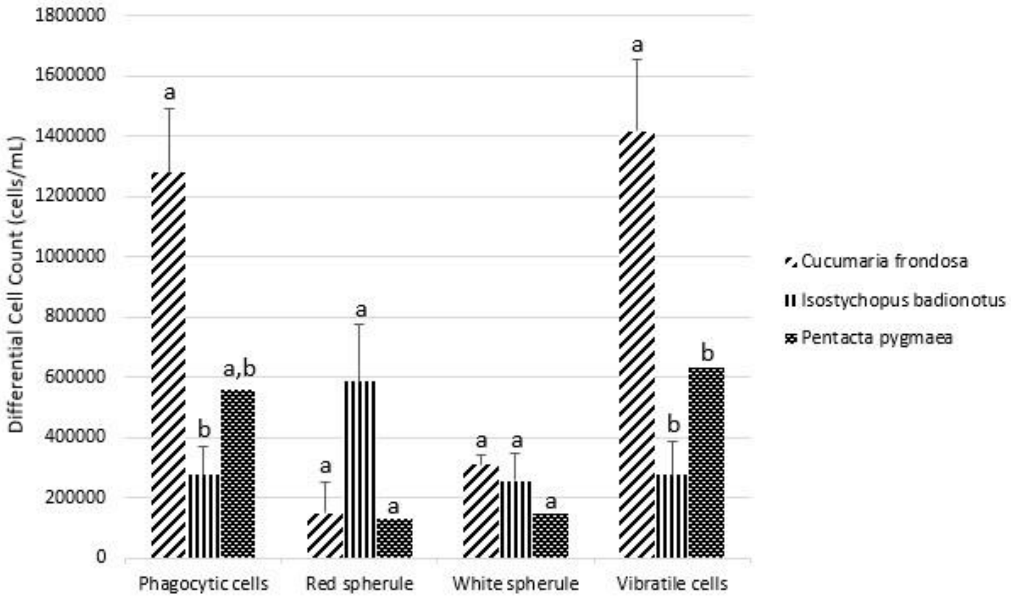

Figure **Error! No text of specified style in document.**.3 Total cell count (cells/mL) of *Cucumaria frondosa* (*C. frondosa*), *Isostychopus badionotus* (*I. badionotus*), and *Pentacta pygmaea* (*P. pygmaea*). The cell counts illustrated are averaged. Different alphabets represent significantly different concentrations of cells among the species (P<0.05).

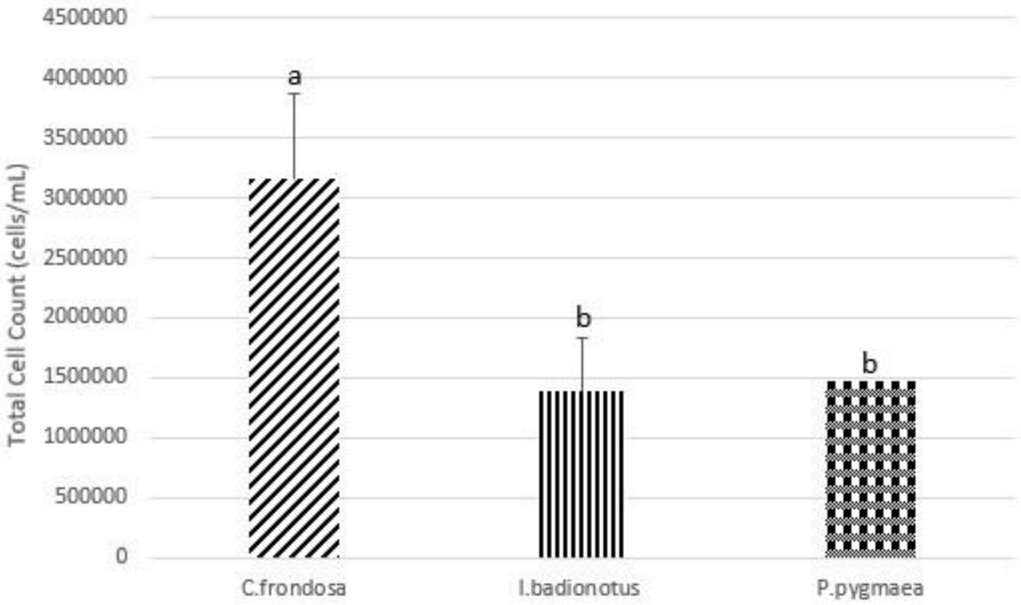

Figure **Error! No text of specified style in document.**.4 Coelomic protein (g/100mL) of *Cucumaria frondosa* (*C. frondosa*), *Isostychopus badionotus* (*I. badionotus*), and *Pentacta*

pygmaea (*P. pygmaea*). The concentrations are illustrated in mean +SEM. Different alphabets represent significantly different concentrations of coelomocyte protein among the species (P<0.05).

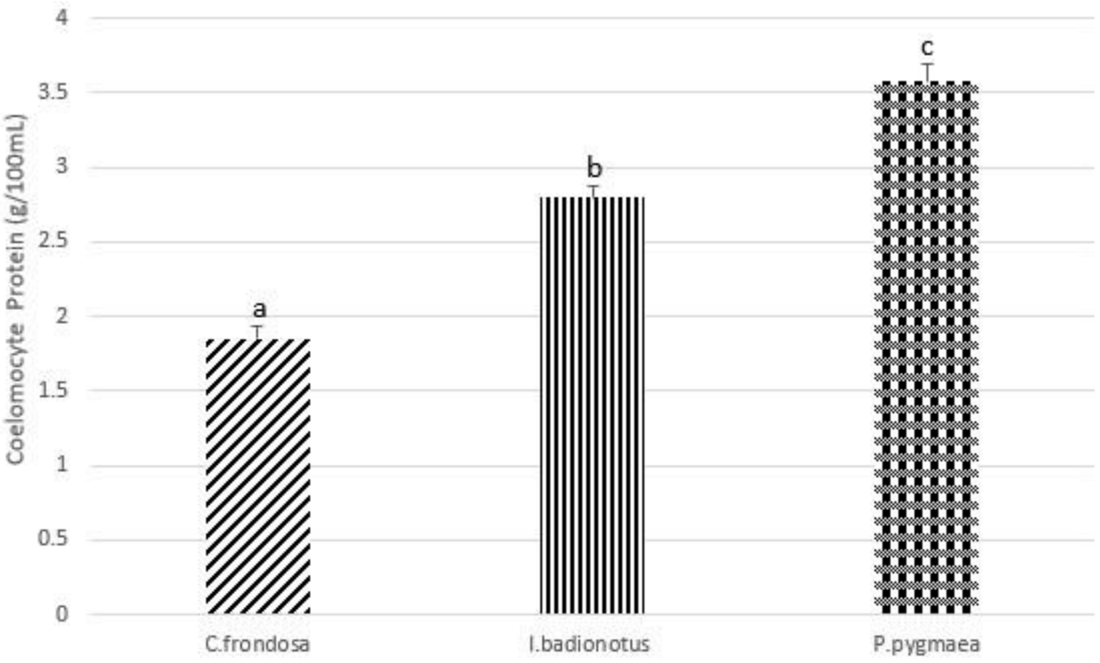

Figure **Error! No text of specified style in document.**.5 Lysozyme activity (absorbance/minute) of *Cucumaria frondosa* (*C. frondosa*), *Isostychopus badionotus* (I. badionotus), and *Pentacta pygmaea* (*P. pygmaea*). The activities are illustrated in mean +SEM. Different alphabets represent significantly different lysozyme activity among the species (P<0.05).

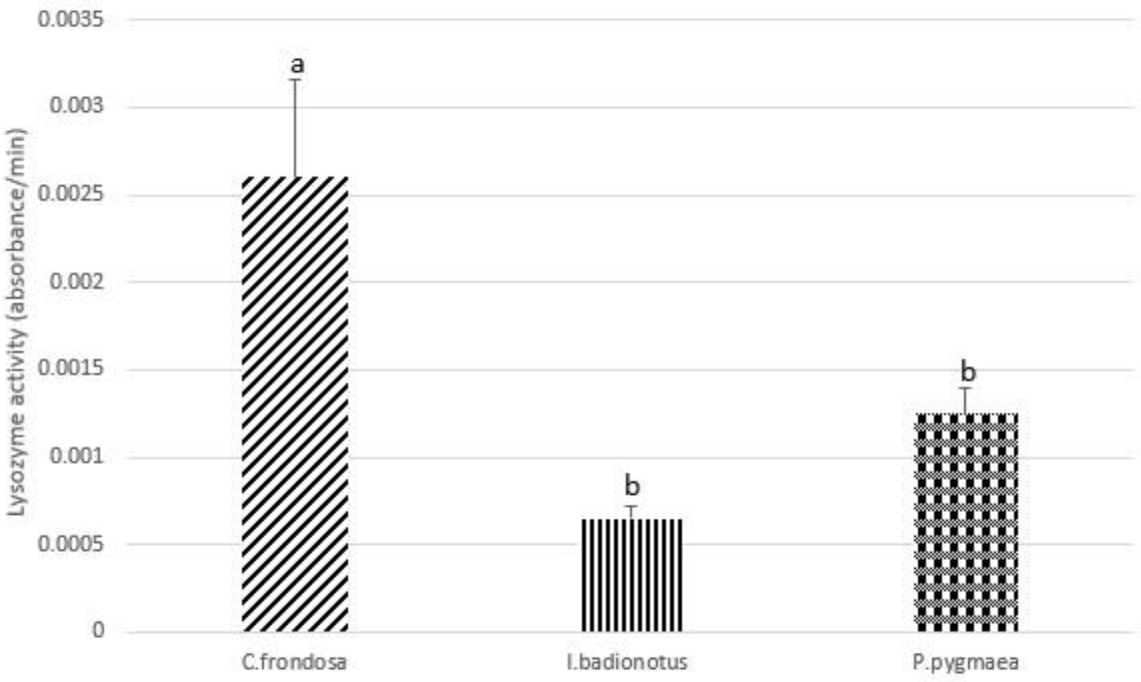

Figure **Error! No text of specified style in document.**.6 Phagocytic capacity (%) of *Cucumaria frondosa* (*C. frondosa*), *Isostychopus badionotus* (*I. badionotus*), and *Pentacta pygmaea* (*P. pygmaea*). The capacities are illustrated in mean +SEM. There were no significant differences among the three species (P<0.05).

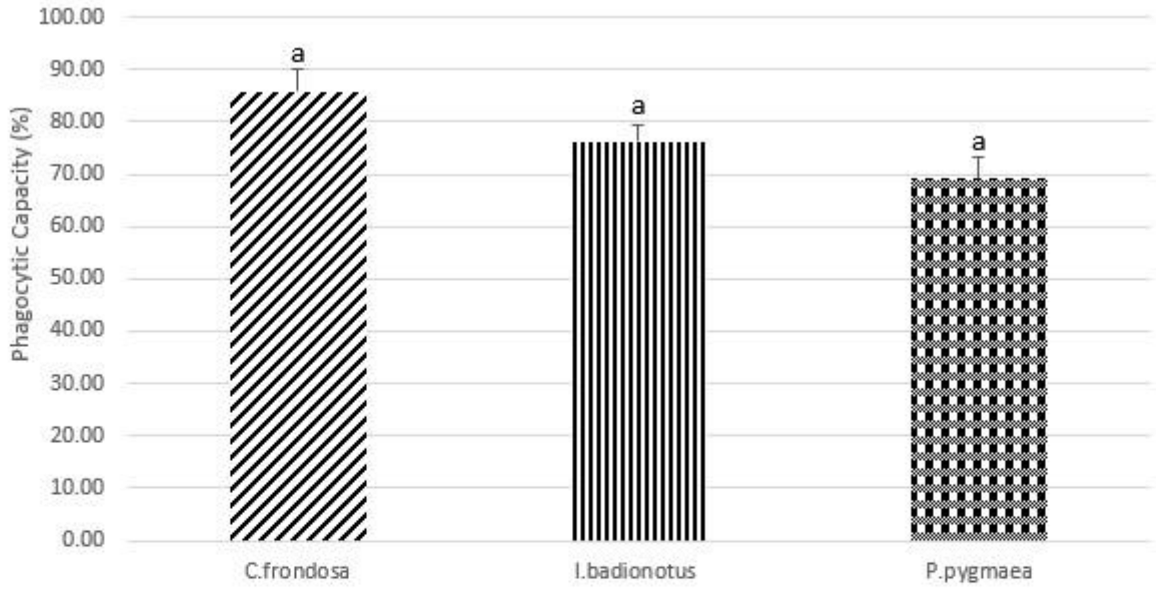

Supplement: Attachment — Submitted filename: PONE-D-23-17088_230707_191110 reviewed .pdf [file pone.0293481.s003.pdf]
