# Supplementary material for: Comparative physiological study of sea cucumbers from eastern waters of United States
Source: PLoS One. 2023 Oct 30;18(10):e0293481. doi: 10.1371/journal.pone.0293481 (PMC10615258; doi:10.1371/journal.pone.0293481)
Supplement: Attachment — Submitted filename: PONE-D-23-17088_reviewer.docx [file pone.0293481.s002.docx]

July 7, 2023

**Comments to the Editor/Author**

Manuscript entitle: “Comparative Physiological Study of Sea Cucumbers from Eastern Waters of United States” [PLOS ONE_PONE-D-23-17088].

The article has some interesting observation and information for Comparative Physiological Study of Sea Cucumbers from Eastern Waters of United States. I am informing you that the manuscript has been recommended and acceptance for publication, but also suggest some suggestion to your manuscript. The Introduction to discussion part gives a good informational and original observation context to the study, but moves abruptly without proper linking to a statement of objectives. The results text should focus on which features of the data sets are of particular very interesting whole manuscript.

1. Authors follow first PLOS ONE general instruction guidelines; carefully check journal formatted of the style.
2. Check the grammar part and plagiarism thoroughly.
3. Authors must incorporate all the corrections which I made in the manuscript.
4. Recent references add me for your future research and outcome of your manuscript.
5. Attention may please be given to the corrections or modifications indicated in the enclosed manuscript while typing the revised manuscript.
6. Whole manuscript thoroughly checks plagiarism part very important and improve the research.
7. Line No. 20, introduction *I. badionotus* should be *Isostychopus badionotus.*
8. Most of the place mentioned reference in number but some other place has been cited author and year? i.e. Xu et al. (2018), Acosta et al. (2021), Yeh et al. (2004), Mumu & Mustafa (2022), Jobson et al. (2021),
9. Figure number 1.1. not available anywhere in the MS, what is the meaning of the figure 2.1, 2.2, 2.3 etc and also figure caption always should be bottom of the figure.

Best Wishes

Thanking you,

**[S. BALAKRISHNAN]**
