# Supplementary material for: Comparative physiological study of sea cucumbers from eastern waters of United States
Source: PLoS One. 2023 Oct 30;18(10):e0293481. doi: 10.1371/journal.pone.0293481 (PMC10615258; doi:10.1371/journal.pone.0293481)
Supplement: Attachment — Submitted filename: Win Responses To Reviewers.docx [file pone.0293481.s005.docx]

August 29, 2023

To:

Professor Mohammed Fouad El Basuini
Academic Editor
PLOS ONE

Manuscript Number: PONE-D-23-17088

Subject: Responses to the reviewers’ comments for Manuscript: PONE-D-23-17088

Dear Editor,

Greetings.

Please review our responses below to the comments made by the reviewers. We believe that upon reviewing our responses, you will be kind enough to accept our manuscript for publication in PLOS ONE.

**Responses to reviewers**

**Reviewers 1 and 2**

- We thank reviewers 1 and 2 for their positive acceptance.

**Reviewer 3**

Comments/suggestions/recommendations:

- p values aren't necessary in the abstract
- remove the word "and" in the keywords
- change "physiological" to physiology in keywords
- "According to research" doesn't add anything to your narrative (e.g. line 101). Either specify the authors or remove the phrase
- line 143. "N" is used for population size, while "n" is used for sample size. Change N to n.
- line 154. remove the word described or say "As described in Yeh et al. [30], the following equation was used to calculate cells per milliliter:"
- In reference to the results section, I believe F typically comes before p

Our response:

- We would like to thank Reviewer 3 for the comments, and we have changed the following recommended comments for the paper.

**Reviewer 4**

Comments/suggestions/recommendations:

- p values aren't necessary in the abstract
- remove the word "and" in the keywords
- change "physiological" to physiology in keywords
- "According to research" doesn't add anything to your narrative (e.g. line 101). Either specify the authors or remove the phrase
- line 143. "N" is used for population size, while "n" is used for sample size. Change N to n.
- line 154. remove the word described or say "As described in Yeh et al. [30], the following equation was used to calculate cells per milliliter:"
- In reference to the results section, I believe F typically comes before p

Our response:

- We would like to thank Reviewer 3 for the comments, and we have changed the following recommended comments for the paper.

| **Reviewers** | **Comments/Suggestions/Recommendation** | **Responses** |
| --- | --- | --- |
| Reviewer #3: | - p values aren't necessary in the abstract - remove the word "and" in the keywords - change "physiological" to physiology in keywords - "According to research" doesn't add anything to your narrative (e.g. line 101). Either specify the authors or remove the phrase - line 143. "N" is used for population size, while "n" is used for sample size. Change N to n. - line 154. remove the word described or say "As described in Yeh et al. [30], the following equation was used to calculate cells per milliliter:" - In reference to the results section, I believe F typically comes before p | **We would like to thank Reviewer 3 for the comments, and we have changed the following recommended comments for the paper.** |
| Reviewer #4: | - Line 154: remove "as described in" | **We appreciate the kind words from Reviewer 4 and we removed the phrase mentioned by Reviewer 4.** |
